# Ordinary Magic in Extraordinary Circumstances: Factors Associated with Positive Mental Health Outcomes for Early Adolescents During the COVID-19 Pandemic

**DOI:** 10.1007/s42844-022-00054-0

**Published:** 2022-01-31

**Authors:** Emma Ashworth, David W. Putwain, Shane McLoughlin, Pooja Saini, Jennifer Chopra, Benjamin Rosser, Catrin Eames

**Affiliations:** 1 School of Psychology, Faculty of Health, Liverpool John Moores University, Liverpool, UK; 2 School of Education, Faculty of Arts, Professional and Social Studies, Liverpool John Moores University, Liverpool, UK

**Keywords:** Adolescence, Protective factors, Mental health, Resilience, COVID-19

## Abstract

The COVID-19 pandemic and associated restrictions have had a negative impact on the mental health and wellbeing of many people worldwide, but this may have been particularly challenging for adolescents. However, there is a paucity of research examining the factors associated with good mental health during this time. The aim of the current study was to identify the protective factors amongst early adolescents in the UK that were associated with better mental health outcomes (internalising and externalising difficulties, and wellbeing) during the first national COVID-19 lockdown. Between September and December 2020, 290 11–14 year olds across North West England completed an online survey consisting of several measures pertaining to experiences of lockdown, and mental health and wellbeing. Hierarchical multiple regression was used to analyse the data. Results indicated that higher participant-rated lockdown experience (the extent to which it was fun, easy, and good) and higher levels of optimism were protective factors for all three outcomes of interest. Greater adherence to government guidance was a protective factor for internalising difficulties and wellbeing only, while family keyworker status was protective for externalising difficulties and wellbeing only. Community and school connection were protective factors for internalising difficulties; family connection and number of parents at home were protective factors for externalising difficulties; and peer support and family knowledge of COVID-19 were protective factors for wellbeing. In summary, the ‘ordinary magic’ of supportive relationships and positive experiences appear to be some of the key factors needed to maintain adolescents’ mental health and wellbeing, and to help them overcome difficulties posed by the COVID-19 pandemic.

Worldwide restrictions to prevent infection during the COVID-19 pandemic have included isolation, social distancing, and school closures (Gov.UK, 2021). Whilst these measures are implemented to reduce the spread of infection and prevent loss of life (British Medical Association, 2020), such measures can have a negative impact on mental health. Risk factors associated with poorer mental health, including uncertainty, anxiety, social isolation, and loneliness, are heightened during pandemics due to both the disease itself and the associated restrictions (Shanahan et al., 2020), and are considered to present particular challenges to adolescents (Branquinho et al., 2020). Evidence suggests that, relative to adults, adolescents are at an increased likelihood of experiencing mental health problems such as depression, anxiety, and heightened stress, including post-traumatic stress, as a result of COVID-19 (Liang et al., 2020; Pascual-sanchez et al., 2020; Raccanello et al., 2020). However, there is currently a paucity of research exploring the promotive and protective factors associated with positive mental health amongst early adolescents during this time. Further knowledge in this area will be useful in enhancing the support that is available for young people, to help ensure they continue to thrive, despite the challenging circumstances.

## Disruption During the COVID-19 Pandemic

Early adolescence (ages 11–14 years) is a key period of development, when young people grow in independence and begin to prioritise relationships with peers over family members (The Lancet Child & Adolescent Health, 2020). Peer relationships are associated with positive wellbeing and adjustment during adolescence (Žukauskiene, 2014), serving as important sources of social support and influence, and helping to shape young people’s behaviour, identity, and attitudes (Telzer et al., 2018). In the UK, repeated school closures during national lockdowns (23rd March–4th July 2020 and 5th November 2020–8th March 2021), alongside community-wide governmental advice such as staying at home, reducing social contact, and closure of leisure and retail businesses, have likely resulted in reduced face-to-face contact with peers and changes to routines that are difficult to adjust to (Lee, 2020). As such, adolescents’ normative developmental experiences are likely to have been disrupted during this time. Whilst the long-term psychological effects of lockdown are unknown, evidence from previous pandemics indicates that loneliness during quarantines is associated with long-term depression and anxiety in adolescents (Sprang & Silman, 2013).

UNESCO (2020) has highlighted the interruption to learning as a key adverse consequence of the pandemic, with lockdown exacerbating existing disparities and health inequalities within the education system. Many young people rely on other school-based provision such as access to teaching facilities, free school meals, and pastoral/wellbeing or safeguarding teams; all of which were substantially reduced during lockdowns (Clemens et al., 2020; The Lancet Child & Adolescent Health, 2020). School closures have meant that many young people with poor mental health or those experiencing other adversities (e.g., poor living conditions, family relations, or poverty) may have missed out on these vital amenities, resulting in detrimental consequences to the most vulnerable young people in our society. For those adolescents who identify with mental health needs, school routines are seen as important coping mechanisms (Lee, 2020).

Early adolescence is a critical period for the onset of mental health difficulties, with 50% of lifetime conditions having their first onset by the age of 14 years old (Kessler et al., 2005). One in seven 11–16 year olds was found to have at least one mental health condition before the COVID-19 pandemic (Sadler et al., 2018). Specifically, internalising disorders (i.e., emotional or inward-facing difficulties such as anxiety and depression) were the most common type of disorder (9%), with girls at an increased risk of experiencing difficulties in this domain (10.9% vs. 7.1%). Conversely, externalising difficulties (i.e., behavioural or outward-facing difficulties such as conduct or hyperactivity disorders) were more common in boys (10.6% vs. 5.7%; NHS Digital, 2018). When combining these existing rates with the heightened adversity and disruption young people have faced during the COVID-19 pandemic, adolescents could be at an even greater risk of developing potentially long-term mental health difficulties.

## Overcoming Adversity

In acknowledging the heightened risk factors and negative impacts of the COVID-19 pandemic and associated restrictions, it is important to highlight the resilience process that some adolescents use to cope and thrive despite adversity (Dvorsky et al., 2020; Wright et al., 2013). The concept of resilience focuses on strengths as well as deficits and can be understood in terms of both risks and protective/promotive factors (Luthar & Cicchetti, 2000). This definition stipulates that in order to demonstrate resilience, children must be flourishing despite exposure to adversity (Masten & Powell, 2003), and considers resilience to be a dynamic process that all children are capable of demonstrating if the right mechanisms are in place (Luthar & Cicchetti, 2000; Masten, 2001). The literature has historically centred on identifying individual characteristics predictive of resilience in children; coping skills, problem solving, self-efficacy, self-regulation, expressiveness, reflectiveness, sense of competence, optimism, autonomy, cognitive skills, temperament, and communication skills have all been identified (Alvord & Grados, 2005; Bonanno & Mancini, 2008; Daniel & Wassell, 2002; Masten, 2001).

However, recent research in the resilience field has indicated a shift in focus from the individual child to the child’s interactions with their environment, and there is increased emphasis on the factors that facilitate the development of wellbeing under stress (Ungar, 2011b, 2012). Wellbeing is defined not simply as the absence of poor mental health, but also the presence of good mental health, whereby individuals with strong general wellbeing are feeling good and functioning well; in other words, they are flourishing (Ng Fat et al., 2017). Resilience work is therefore moving away from the study of vulnerable children, to a focus on the socio-ecological factors that operate at multiple levels to promote good wellbeing; thus, family and community factors are also integral (Landau, 2010). Furthermore, in keeping with Bronfenbrenner’s ecological systems theory (1977), it is thought that these levels interact with and impact one another (Masten, 2007). Thus, Goldstein and Brooks (2013) advocate that resilience research should centre on the interaction between the child and their social environment. Ungar (2011a) further developed this by suggesting that resilience could be understood as the child’s ability to access the resources they need from the community, in order to establish and maintain their wellbeing in the face of risk or adversity. Masten (2001) posited that these resources, known as protective factors, come from ‘everyday’ elements of their environment, such as having trusted friendships and supportive family; in other words, Masten suggested protective factors to be ‘ordinary magic’ (Masten, 2001).

Whilst optimism is closely aligned with resilience, it is considered distinct — defined as a stable personality trait, with generalised positive outcome expectancies (Scheier & Carver, 1985; Snyder et al., 1991), thought to be most relevant in situations with little scope for personal control (Alarcon et al., 2013; Gallagher & Lopez, 2009). Optimism has been found to be positively related to psychological wellbeing, and negatively related to depression and anxiety (Alarcon et al., 2013); with higher levels of optimism associated with better subjective wellbeing in times of adversity (Carver et al., 2010). Optimism has been positively associated with coping styles in light of stressful events, demonstrating flexibility of adopting problem-focused coping with controllable stressors and emotion-focused coping with uncontrollable stressors as the situation demands (Solberg Nes & Segerstrom, 2006).

Whilst many promotive and protective factors (individual, social, and environmental attributes that are associated with positive youth development; Fergus & Zimmerman, 2005) have previously been established in the extant evidence base, there is limited research explicitly examining the protective factors during the COVID-19 pandemic and its associated restrictions, such as school closures. Given the change in social and familial circumstances for young people because of COVID-19-related restrictions, the resources typically drawn from in terms of resilience may also be limited. Dispositional optimism may therefore act as a protective factor when community factors are disrupted. There is also sparse research exploring the specific COVID-19-related factors that may boost adolescents’ wellbeing during the pandemic, such as family shielding (protecting family members defined on medical grounds as extremely clinically vulnerable to COVID-19) or keyworker status (people working within health and social care, education and childcare, public safety and national security, transport, utilities, and communication, food and other necessary goods, financial services, and key national and local government), and individuals’ understanding of and level of worry regarding COVID-19.

## Resilience During the COVID-19 Pandemic

Just as we need to understand which young people are at increased risk of mental health difficulties during this pandemic, it is also vital we understand the factors that help adolescents to thrive. Unification of evidence relating to both risk and protective factors provides a more holistic picture of what is happening in young people’s lives (Wood & Tarrier, 2010). Furthermore, an understanding of the effective protective factors associated with positive mental health and wellbeing can advance our understanding of how young people respond to crises and can help inform the services and resources offered (Dvorsky et al., 2020).

Emerging evidence suggests that optimism is important for promoting resilience during the COVID-19 pandemic (Xie et al., 2020), with 12–18 year olds in China who report higher levels of optimism for the COVID-19 pandemic reporting lower levels of anxiety and depression symptoms (Zhou et al., 2020). Furthermore, older adolescents aged 16–19 years in a qualitative study in the UK reported adopting a positive outlook (e.g., remaining hopeful and optimistic for the future) as an intentional coping strategy for promoting good wellbeing during the COVID-19 pandemic (Demkowicz et al., 2020), with similar themes regarding intentional coping strategies reported amongst 16–24 year olds in Portugal (Branquinho et al., 2020). Repeated lockdown phases (that is, strict government-led restrictions of limited social contact, social movement not allowed between social households and school, leisure and non-essential business closures) indicate shifts in attitudes towards lockdown experiences, with 2438 13–25 year olds surveyed in January 2021 in the UK reporting having implemented and established coping mechanisms and routines they had learnt from two earlier periods of strict lockdown restrictions to support their mental health, and 79% believing their mental health will improve once restrictions are lifted (YoungMinds, 2020). However, most of these protective factors are individual-level attributes, and so the social and environmental (and specifically the pandemic-related) factors that are associated with better adolescent mental health outcomes are currently unknown.

## The Current Study

Whilst evidence continues to emerge for the effects of the COVID-19 pandemic and associated restrictions on the mental health and wellbeing of young people, most research has focused specifically on the negative outcomes. Indeed, evidence so far suggests that the pandemic has put young people at increased risk of experiencing poor mental health (e.g., Loades et al., 2020). Thus, more research is needed regarding the protective factors that counteract this risk and are associated with good mental health outcomes in young people, and in particular, the protective factors related specifically to the pandemic. Furthermore, most COVID-19-related research has investigated the impact on adults or older adolescents (16–19 years e.g., Demkowicz et al., 2020; Pascual-sanchez et al., 2020); less evidence exists regarding the experiences of younger adolescents. This is particularly important given that they are at a critical stage in their development and a point of heightened vulnerability to mental health difficulties (Kessler et al., 2005).

Thus, this study aimed to examine not only the psychological protective factors, but also the social and environmental factors, that were associated with greater mental health outcomes during the first lockdown in early adolescents in the UK. Specifically, we aimed to identify the factors within the home, school, peers, and the community, as well as the factors related to the pandemic specifically (e.g., adherence to guidelines, living with key workers) that were associated with better mental health outcomes, namely internalising difficulties (inward facing difficulties e.g., emotional problems), externalising difficulties (outward facing difficulties e.g., conduct problems), and general wellbeing, during this time.

## Methods

### Participants and Recruitment

Five secondary schools participated in the study, three were coeducational and two were single sex (one boys’ school and one girls’ school). The single sex schools were also academically selective^1^, and the girls’ school was fee paying. Two hundred and ninety pupils in years 7–9 (the first 3 years of secondary education) participated, aged between 11 and 14 years (*M* = 11.95; *SD* = 0.85). Of the participants, 52.8% identified as male and 45.2% as female, with the remaining 2% identifying as ‘other’ or indicating that they preferred not to say. Sixteen percent reported that they were in receipt of free school meals (FSM; a proxy for belonging to a low-income household). Other demographic data is presented in Table 1. The sample is broadly in line with the national average for pupils of this age in terms of proportion eligible for FSM (17.3%) and belonging to a Black or Minority Ethnic (BAME) group (32.3%) (Department for Education, 2021b).

**Table 1:** Demographic data

Demographic	%
Gender	
Male	52.8
Female	45.2
Other/prefer not to say	2.0
Ethnicity	
White	76.9
Asian/Asian British	8.8
Mixed Ethnicity	7.5
Chinese/Chinese British	2.0
Black/Black British	1.0
Another ethnic group	2.0
Sexuality	
Heterosexual	79.9
LGBTQIA+	9.5
Prefer not to say	10.2
Religion	
Christianity	32.7
Islam	8.2
Hinduism	1.4
Receiving free school meals	16

An a priori sample size calculation, performed using the G*Power software v. 3.1.9.2 (Faul et al., 2007, 2009), showed a minimum sample of size of 208 participants would be required to detect a moderate effect size (*f*^2^ = 0.15) at standard alpha (*p* < 0.05) and power (0.95) values for 17 predictor variables. There were 12.8% of values missing in the data. In order to establish any systematic variation in patterns of missingness, an omnibus test for missing completely at random (MCAR) was conducted using Little’s test. Little’s test was not statistically significant, *χ*^2^ (1266) = 1333.54, *p* = 0.09, indicating MCAR can be assumed. Accordingly, missing data were handled using an expectation-maximisation (EM) algorithm in the SPSS v.25 software. EM is preferred to listwise and pairwise deletion which can result in biased parameter estimates (Graham, 2012).

### Measures

The survey consisted of three parts: Part 1 asked participants a series of questions relating to their demographic characteristics, Part 2 included questions about adolescents’ experiences of lockdown, and Part 3 presented a series of measures pertaining to participants’ mental health and wellbeing.

#### Demographics

Part 1 presented a series of questions regarding participants’ age, gender identity, ethnic group, religion, sexuality, and FSM status. All questions were optional and offered a ‘prefer not to say’ response.

#### Lockdown Factors

Part 2 contained a series of questions designed for this study, asking about participants’ experiences of COVID-19 and what their lockdown looked like. Questions asked about their home and who they were living with (e.g., *‘who is looking after you?’; 1 parent/2 parents/carers/grandparents/other family member/other*), including if any household members were shielding or were key workers (*yes/no/don’t know/prefer not to say*). There were also questions asking participants’ to rate theirs and their family members’ perceived knowledge level regarding COVID-19 (*1 = poor; 7 = good)*, and the extent to which they were following the guidance (*1 = not at all; 7 = completely*). Six items pertained to ‘fear of COVID-19’, asking participants to rate the extent to which they were worried about themselves or their family member becoming unwell with COVID-19 (e.g., ‘*if my friends and family were to develop COVID-19 they would suffer badly from it’; 1 = strongly disagree, 5 = strongly agree)*; the internal consistency was *α* = 0.84. Participants were also asked about their experiences of lockdown. Participants were presented with three items relating to lockdown and were asked to rate their experiences of each on five-point Likert scales (*very bad–very good; very hard–very easy; very boring–very fun*). Scores for these items were summed, to form an overarching ‘lockdown experience’ variable, with higher scores indicating a better lockdown experience (the internal consistency was *α* = 0.80).

#### Mental Health Difficulties

Me and My Feelings (M&MF; Deighton et al., 2013) was used to measure mental health difficulties. M&MF is a brief, 16-item school-based self-report measure of child mental health, covering two broad domains: internalising difficulties (e.g., emotional problems) and externalising difficulties (e.g., behavioural problems). Statements are provided (e.g., *I feel lonely; I lose my temper*), and young people are asked to rate the extent to which they feel each statement represents them on a three-point Likert scale (*never/sometimes/always*). The first 10 items comprise the internalising difficulties subscale, whilst the remaining six form the externalising difficulties subscale. Scores are summed for each subscale, with higher scores indicating higher levels of difficulties. Internal consistency in the context of the present data was *αs* = 0.77–0.80. Psychometric properties are well reported, including previously established construct, convergent, and discriminant validity, and the measure has been validated for use with children aged 8 years and over (Deighton et al., 2013; Patalay et al., 2014).

#### Wellbeing

The Short Warwick-Edinburgh Mental Wellbeing Scale (SWEMWBS; University of Warwick & University of Edinburgh, 2008) was utilised as a measure of mental wellbeing. SWEMWBS is a seven-item self-report measure, consisting of a series of positively-worded statements about thoughts and feelings (e.g., *I have been feeling relaxed*). Participants are asked to rate each statement on a five-point Likert scale (*1 = none of the time, 2 = rarely, 3 = some of the time, 4 = often, and 5 = all of the time*) that best describes their experiences over the last two weeks. Scores are summed, with higher scores indicative of higher positive mental wellbeing. The SWEMWBS is recommended for use with secondary school pupils ((Evidence Based Practice Unit, 2018; Ng Fat et al., 2017)) and has established convergent and construct validity (Ringdal et al., 2018). Internal consistency for the current study was *α* = 0.88.

#### Resilience

##### Sources of Support

Four subscales from the Student Resilience Survey (SRS; Lereya et al., 2016) were used to measure participants’ perceptions of factors at the individual-level, as well as those embedded in the environment. Specifically, the family connection (four items), peer support (11 items), community connection (four items), and school connection (four items) subscales were used. Respondents are presented with a series of statements (e.g., *at school there is an adult who really cares about me*), and they are asked to rate the extent to which each statement fits them best on a five-point Likert scale (*1 = never; 5 = always*). Scores are summed for each subscale, with higher scores indicating greater levels of support in each domain. Psychometric properties include criterion validity, and validated for use in children aged 11 years and over (Lereya et al., 2016). Internal consistency in the current study was *α* = 0.80–0.93.

##### Optimism

The revised Life Orientation Test (LOT-R; Herzberg et al., 2006) was used as a measure of optimism. The LOT-R is a 10-item self-report measure designed to assess individual differences in generalised optimism versus pessimism. Participants are presented with a series of statements (e.g., *in uncertain times, I usually expect the best*; *if something can go wrong for me, it will*), and are asked to rate the extent to which they agree on a five-point Likert scale (*strongly disagree/disagree/neutral/agree/strongly agree*). Scores are summed, with higher values indicating higher levels of optimism. The LOT-R has successfully been utilised with secondary school aged children and has reported discriminant validity (Creed et al., 2002; Wong & Lim, 2009). In the current study, internal consistency was *α* = 0.60–0.78.

### Design and Procedure

The current cross-sectional study utilised quantitative survey data collected as part of the Adolescents’ Lockdown-Induced Coping Experiences (ALICE) study. The ALICE study was conducted in the North West of England with five secondary schools, between September and December 2020, following the first UK lockdown. Secondary schools were recruited to participate via social media, and through existing networks and connections. Schools sent information sheets to the parents/carers of all pupils in years 7–9, along with a link to an online survey, consisting of a suite of measures exploring young people’s mental health and wellbeing during the COVID-19 pandemic, and their experiences of lockdown. If the parents/carers consented to their child taking part, they were asked to provide them with the survey link. Informed assent was sought from the young people, who were asked to tick a box at the beginning of the survey if they consented to taking part. The ALICE study received ethical approval from the institutional Research Ethics Committee (Ref: 20/NSP/037).

### Analytic Strategy

Hierarchical multiple regression analyses were conducted to establish how much variance in the three outcome variables of interest (internalising difficulties, externalising difficulties, and wellbeing) could be accounted for by the predictor variables (lockdown factors and resilience factors), after controlling for demographic characteristics (school, gender, age, and FSM status). For each of the three models, demographic variables were added in step 1 in order to control for their effects, lockdown factors added in step 2 to establish the unique variance explained by predictors relating specifically to the COVID-19 pandemic, and finally resilience factors added in step 3.

## Results

### Descriptive Statistics

Descriptive statistics are presented in Table 2. Participants generally rated themselves as having high levels of support from home, school, the community, and their peers, with support from home rated highest. Levels of wellbeing were in line with population norms for SWEMWBS (Ng Fat et al., 2017). Mean scores for optimism, internalising difficulties, and externalising difficulties fell around the mid-way point for all three variables (3.3/5, 1.7/3, 1.5/3 respectively). The intra-class correlations (ICCs) represent the proportion of variance attributable to the school that participants were recruited from. These were small (ICCs < 0.05) with the exception of the number of siblings and home, family connection, and community connection. As the number of upper-level units (i.e., schools) was too small to warrant a multi-level model, we accounted for this variance by including school as a covariate, along with demographic variables, in the first step of the regression model.

**Table 2:** Descriptive statistics

	Mean	*SD*	Observed ranges	Skewness	Kurtosis	ICCs
**PART 2**						
Experience of lockdown	2.93	0.94	1–5	0.08	−0.33	0.02
Fear of COVID-19	3.75	1.36	1–7	0.24	−0.59	0.01
Number of parents at home	1.85	0.36	1–2	−1.98	1.95	0.01
Number of siblings at home	1.30	1.13	0–8	2.28	9.84	0.05
Personal knowledge of COVID-19	5.53	1.27	1–7	−0.48	−0.44	0.01
Family knowledge of COVID-19	6.17	1.00	1–7	−1.08	0.68	0.02
Following government guidance	5.77	1.16	2–7	−0.77	0.01	0.01
**PART 3**						
Resilience: family connection	4.55	0.59	1.25–5	−2.07	5. 43	0.05
Resilience: school connection	3.79	0.91	1–5	−0.55	−0.10	0.03
Resilience: community connection	4.15	1.05	1–5	−1.02	0.52	0.07
Resilience: peer support	4.11	0.83	1.36–5	−1.02	0.52	0.01
Resilience: optimism	3.30	0.57	1.6–4.9	−0.21	0.36	0.02
**OUTCOMES**						
Internalising difficulties	1.69	0.42	1–2.8	0.42	2.33	0.01
Externalising difficulties	1.49	0.40	1–2.83	0.92	0.82	< 0.01
Wellbeing	3.49	0.72	1.14–5	−0.38	0.29	< 0.01

### Bivariate Correlations

Bivariate correlations are presented in Table 3. Levels of optimism had the strongest relationship with all three outcome variables. Regarding lockdown-related factors, the majority were associated with levels of internalising difficulties, externalising difficulties, and wellbeing. Number of parents at home and having a family member shielding at home, as well as the number of siblings at home, were associated with internalising difficulties only, and fear of COVID-19 with wellbeing only. Family connection was the resilience factor most strongly associated with lower levels of internalising and externalising difficulties, whilst peer support was the resilience factor most strongly associated with higher levels of wellbeing.

**Table 3:** Bivariate correlations

	1.	2.	3.	4.	5.	6.	7.	8.	9.	10.	11.	12.	13.	14.	15.	16.	17.
1. Experience	—	−0.11	0.06	0.06	0.01	−0.12	−0.03	0.07	0.17**	0.23**	0.08	0.11	0.13*	0.28**	−0.46**	−0.34**	0.37**
2. Fear		—	0.07	0.13*	−0.14*	0.22**	−0.03	−0.04	−0.10	−0.13*	0.01	−0.06	−0.13*	−0.22**	0.23**	0.16**	−0.10
3. *N* Parents			—	0.14*	0.01	−0.03	−0.04	0.01	−0.04	0.02	0.06	0.01	0.02	0.11	−0.09	−0.11	0.05
4. *N* Siblings				—	−0.12*	0.08	−0.15*	−0.29**	−0.09	−0.25**	−0.13*	−0.13*	−0.19**	−0.04	0.08	0.13*	−0.12**
5. Keyworker					—	−0.04	0.06	0.11	0.11	0.07	0.11	−0.04	0.08	0.11	−0.12*	−0.15*	0.20**
6. Shielding						—	0.07	0.18**	0.02	0.73	0.03	−0.002	0.01	−0.06	0.07	0.10	−0.03
7. Personal knowledge							—	0.63**	0.29**	0.29**	0.24**	0.11	0.19**	0.21**	−0.14*	−0.18**	0.23**
8. Family knowledge								—	0.26**	0.37**	0.22**	0.15*	0.25**	0.24**	−0.19**	−0.17**	0.31**
9. Guidance									—	0.38**	0.24**	0.19**	0.22**	0.21**	−0.32**	−0.26**	0.35**
10. Home support										—	0.43**	0.60**	0.54**	0.52**	−0.49**	−0.43**	0.55**
11. School support											—	0.46**	0.51**	0.31**	−0.35**	−0.28**	0.41**
12. Community support												—	0.49**	0.38**	−0.39**	−0.28**	0.42**
13. Peer support													—	0.49**	−0.44**	−0.28**	0.58**
14. Optimism														—	−0.68**	−0.43**	0.72**
15. Internalising															—	0.49**	−0.71**
16. Externalising																—	−0.50**
17. Wellbeing																	—

*p < 0.05. **p < 0.01. ***p < 0.001

### Hierarchical Regression Analyses

#### Internalising Difficulties

For Model 1 (internalising difficulties) a significant model was identified in step 3 (Table 4), *F* (18,271) = 22.37, *p* < 0.001. The *R*^*2*^ indicates the predictors in the model accounted for approximately 60% of the variance in internalising difficulties, indicative of a large effect size (Cohen, 1992). After controlling for demographic factors, two lockdown factors emerged as significant predictors, lockdown experience and the extent to which participants followed government guidance, with more positively rated lockdown experiences (*β* = −0.26, *p* < 0.001) and higher levels of adherence to guidance (*β* = −0.12, *p* = 0.002) associated with lower levels of internalising difficulties. Lockdown experience was a stronger predictor of internalising difficulties than guidance adherence. Optimism emerged the largest predictor in step 3, with higher levels of optimism associated with lower levels of internalising difficulties (*β* = −0.48, *p* < 0.001). In addition, higher levels of community (*β* = −0.09, *p* = 0.04) and school (*β* = −0.08, *p* = 0.04) connection were associated with fewer internalising difficulties.

**Table 4:** Hierarchical multiple regression models (step 3)

	Internalising difficulties			Externalising difficulties problems			Wellbeing		
	Δ*R*^*2*^	*β*	*sr*	Δ*R*^*2*^	*β*	*sr*	Δ*R*^*2*^	*β*	*sr*
*Step 1: Demographics*	0.043**			0.028*			0.052**		
School		−0.025	−0.037		0.024	0.027		0.017	0.028
Gender		0.047	0.071		0.038	0.045		−0.033	−0.057
Age		0.010	0.015		0.019	0.037		−0.005	−0.008
FSM		0.018	0.026		0.017	0.022		−0.049	−0.079
*Step 2: Lockdown factors*	0.292***			0.207***			0.264***		
Experience of lockdown		−0.260***	−0.345		−0.227***	−0.241		0.166***	0.252
Fear of COVID-19		0.060	0.087		0.026	0.029		0.103**	0.165
Number of parents at home		−0.025	−0.038		−0.087*	−0.102		−0.016	−0.027
Number of siblings at home		0.023	0.033		0.076	0.084		−0.019	−0.030
Personal knowledge of COVID-19		0.038	0.044		−0.097	−0.087		−0.033	−0.043
Family knowledge of COVID-19		0.013	0.015		0.079	0.069		0.081*	0.101
Following government guidance		−0.122**	−0.168		−0.060	−0.065		0.122***	0.185
Family member shielding		0.005	0.007		0.041	0.046		−0.027	−0.044
Family member keyworker		−0.024	−0.036		−0.079*	−0.091		0.096**	0.157
*Step 3: Resilience factors*	0.263***			0.094***			0.356***		
Family connection		−0.024	−0.024		−0.196**	−0.150		0.016	0.018
School connection		−0.085*	−0.098		−0.098	−0.021		0.055	0.071
Community connection		−0.078*	−0.097		−0.024	−0.086		0.039	0.054
Peer support		−0.051	−0.058		0.055	−0.049		0.213***	0.262
Optimism		−0.475***	−0.493		−0.200**	−0.181		0.500***	0.550
*Total R*	0.598			0.329			0.672		

*Note*. Standardised regression coefficients reported from Step 3 of the model. *p < 0.05. **p < 0.01. ***p < 0.001

#### Externalising Difficulties

For Model 2 (externalising difficulties), a significant model was identified in step 3, *F* (18,271) = 7.39, *p* < 0.001. The *R*^*2*^ indicates the predictors in the model accounted for approximately 33% of the variance in externalising difficulties, indicative of a large effect size. After controlling for demographic factors, three lockdown factors significantly predicted externalising difficulties. A more positively rated lockdown experience (*β* = −0.23, *p* < 0.001), having a higher number of parents at home (*β* = −0.09, *p* < .05), and having a family member who was a keyworker (*β* = −0.08, *p* = 0.04) were associated with lower levels of externalising difficulties. Furthermore, two resilience factors, family connection and optimism, were also significant predictors, with higher levels of family connection (*β* = −0.20, *p* = 0.004) and optimism (*β* = −0.20, *p* = 0.001) associated with lower levels of externalising difficulties.

#### Wellbeing

For Model 3 (wellbeing), a significant model emerged in step 3, *F* (18,289) = 30.85, *p* <. 0001. The *R*^2^ indicates the predictors in the model accounted for 67% of the variance in wellbeing, indicative of a large effect size. After controlling for demographic factors, five lockdown factors emerged as significant predictors: lockdown experience, fear of COVID-19, family knowledge of COVID-10, having a family member who was a keyworker, and the extent to which guidance was followed. A more positively rated lockdown experience (*β* = 0.17, *p* < 0.001), higher levels of concern about COVID-19 (*β* = 0.10, *p* = 0.002)^2^, higher family knowledge of COVID-19 (*β* = 0.08, *p* = 0.03), a family member who was a keyworker (*β* = 0.10, *p* = 0.003), and higher levels of adherence to the government guidance (*β* = 0.12, *p* = 0.002), were associated with higher levels of wellbeing. Two resilience factors, peer support and optimism, emerged as significant predictors in step 3, with higher peer support (*β* = 0.21, *p* < 0.001) and optimism (*β* = 0.50, *p* < 0.001) associated with higher levels of wellbeing. Optimism was a stronger predictor of wellbeing than peer support.

## Discussion

The present study aimed to identify the individual, social, environmental, and COVID-19 specific protective factors associated with better mental health outcomes (namely internalising and externalising difficulties, and wellbeing) for 11–14 year olds following the first UK lockdown. Results indicated that higher participant-rated lockdown experience (perceptions of the extent to which it was fun, easy, and good) and higher levels of optimism were protective factors for all three outcomes of interest. Greater adherence to government guidance was a protective factor for internalising difficulties and general wellbeing. Higher levels of community and school connection were also protective factors for internalising difficulties only. Stronger family connection and number of parents at home were protective factors for externalising difficulties only. Having a family member who was a key worker was a protective factor for externalising difficulties and general wellbeing. Finally, stronger peer support and greater family-level knowledge of COVID-19 were protective factors for general wellbeing only.

It is particularly noteworthy that despite the significant impact COVID-19 has had on the daily lives of adolescents, mean scores for lockdown experience ratings were approximately three out of five, indicating that, generally, adolescents in this study did not find it to be a completely negative experience, a potentially reassuring sign. Indeed, research conducted during the COVID-19 pandemic suggests that on average there has been wide-spread resilience in response to the pandemic (Mental Health Foundation, 2020). However, it is important to bear in mind that this score is the average, and some young people in this study reported a very challenging lockdown. Given that lockdown experience was a protective factor across all three aspects of mental health and wellbeing, this is particularly concerning. Thus, it is vital that, moving forward, young people who experienced a difficult lockdown are identified and receive appropriate intervention and support (Ungar, 2011a). Furthermore, should any further lockdowns be required, it will be of upmost importance that these adolescents are appropriately supported, and their lockdown experience improved as far as possible.

Schools are typically considered to be a valuable location for the early identification of children experiencing mental health difficulties and the implementation of mental health interventions (e.g., promoting mental health literacy, social and emotional wellbeing, coping skills; Caan et al., 2015; Vostanis et al., 2013). It is now recognised that all schools have a responsibility for supporting the mental health of their pupils, particularly following the recent Government Green Paper recommending that all English schools appoint a designated mental health lead (Department of Health & Department for Education, 2017). However, prioritising mental health conflicts with more recent governmental concerns regarding pupils’ academic progress, which places schools under considerable pressure to ensure their pupils ‘catch up’ following the school closures (e.g., Department for Education, 2021a). Thus, schools may find themselves in a position where they are in a conundrum regarding whether to reach targets for academic progress or prioritise ensuring the mental wellbeing of their pupils. Nevertheless, given the importance of peer support to adolescents’ general wellbeing identified in the present study (and in previous research e.g., Žukauskiene, 2014), it appears that, particularly for those who have had a difficult lockdown, allowing time in school for adolescents to socialise with their friends and build relationships may be invaluable in counteracting the negative experiences of lockdown. In case any further lockdowns occur, it will also be vital that, as far as possible, schools help to ensure their pupils’ experiences of learning in lockdown are positive; for instance, through the provision of pastoral support and frequent contact with teachers. Qualitative research highlights the importance of teachers, with adolescents emphasising the value of having access to teachers for their learning and wellbeing during school closures (Ashworth et al., 2021).

More broadly, government policy must prioritise the provision of effective support and resources for young people who are experiencing, or who have previously experienced, difficult lockdowns. This may be in the form of strategies that can directly improve a young person’s lockdown (e.g., providing support to families experiencing poverty, or technology for adolescents to access schoolwork), or the availability of resources and support services (e.g., adolescent mental health services) that can help to mitigate the longer-term impact of any negative experiences. Findings from the Mental Health Foundation’s (2020) research echo this need, calling on the government to speed up the roll out of evidence-informed psychotherapeutic digital mental health interventions, implement trauma-informed therapies, and provide safe places for social connection and interaction in the community.

Regarding optimism, greater levels of optimism were associated with greater wellbeing, and less internalising and externalising difficulties. These findings highlight optimism as a personality trait which is associated with resilience and wellbeing, often in the face of a lack of personal control, where dispositional optimism is considered a stress buffer on mental health in adolescents (Lai, 2009). The COVID-19 pandemic, and in particular the first lockdown phase considered in the current study, presented a time of uncertainty, and results suggest that those adolescents who have a more optimistic stance are more likely to experience fewer psychological difficulties and greater wellbeing, thus potentially mitigating the psychological impact of COVID-19 (Muñoz-Fernández & Rodríguez-Meirinhos, 2021). This is consistent with findings that suggest optimism mediates the relationship between stress related to COVID-19 and psychological difficulties (Arslan et al., 2020; Arslan & Yıldırım, 2021). Whilst in this study dispositional optimism is explored, learned optimism can also be applied as a useful intervention in enhancing wellbeing and a movement towards flourishing for the individual (Seligman, 2006).

Another noteworthy finding from the present study is the importance of relationships; specifically, the association between strong family connection and lower levels of externalising difficulties, and strong peer support and improved wellbeing. This finding is consistent with qualitative research with older adolescents that suggests family disputes and tension could be a source of difficulty during lockdowns, whereas keeping in touch with peers was considered beneficial (Demkowicz et al., 2020). The association between family relationships and adolescents’ mental health is perhaps unsurprising given that the vast majority of participants were likely solely with their family for prolonged periods during lockdown, and the existing evidence highlighting the important role of family relationships. For instance, even in the absence of risk, well-structured home environments and warm relationships within the family are important for positive development (Lereya et al., 2016), but having a supportive family has been found to be particularly valuable for children trying to cope with stressful experiences (Bai & Repetti, 2015; Masten & Shaffer, 2006), as is the case at present for many young people.

However, the relationship between stronger family connection and lower levels of externalising difficulties specifically is interesting. Previous research has found stronger associations between family functioning and children’s externalising difficulties than internalising difficulties (Sikora et al., 2013), although it tends to be adolescents with higher levels of externalising difficulties who later experience worse family functioning, not vice versa (Mastrotheodoros et al., 2020). Unfortunately, due to the cross-sectional nature of the current study, we cannot identify the direction of the relationship found. Furthermore, previous studies have focused on family functioning; less research has looked at family *connection*, and so the direction of the relationship remains unclear.

Nevertheless, the findings from the current study, combined with existing evidence, still highlight the importance of fostering good relationships with family members, in order to effectively promote adolescents’ mental health during the pandemic. Various strategies exist, such as family support programs which aim to improve parent wellbeing and parenting, and in turn improve adolescent mental and behavioural health (Laird & Kuhn, 2014), or the provision of family support workers (FSWs) for those identified as at-risk. FSWs can help to strengthen parents’ and children’s social supports and coping skills through the provision of a range of behavioural and parent-training interventions, in order to prevent the escalation of more severe difficulties (Window et al., 2004). Alternatively, parenting classes may provide a less resource-intensive and more readily available option that can be implemented in the community, whilst still strengthening family connections (Brotman et al., 2011).

Outside of the family, peer support was associated with higher levels of general wellbeing. Early adolescence is a crucial period of social development; young people spend more time building relationships outside of the family, and peers become increasingly important in terms of identity formation, fostering independence, and the development of social skills (Shanahan et al., 2007). Thus, given the circumstances of lockdown, whereby young people could not socialise with their peers face-to-face, maintaining strong peer support may have been more important than ever for promoting adolescents’ wellbeing. Furthermore, early adolescence is a time where peers are increasingly relied upon for social support (Telzer et al., 2018). Previous research in the field of help-seeking suggests that young people have a preference for informal sources of support, such as peers, if they are experiencing mental health difficulties (Radez et al., 2020; Rickwood et al., 2005), which may also explain why stronger peer support was beneficial during the coronavirus lockdowns.

Other research has highlighted similar concerns regarding adolescents’ peer groups, with young people reporting they found maintaining relationships during lockdown difficult (Ashworth et al., 2021; Demkowicz et al., 2020), and 41% of 8–24 year olds saying they are lonelier than pre-pandemic (Youth Sport Trust, 2020). As mentioned previously, allowing young people to spend time with peers and develop their relationships on their return to school will be of upmost importance. Indeed, a panel of child mental health experts have already written to the government, urging them to prioritise children’s social and emotional wellbeing when re-opening schools and emphasising the importance of play (Cartwright-Hatton et al., 2020). Strategies such as peer support initiatives may also be beneficial in schools. These can include approaches such as peer tutoring or mentoring, peer counselling, befriending, or buddy systems. Previous research suggests peer support initiatives cannot only promote wellbeing and positive mental health but also facilitate appropriate and quality access to help and signposting for further support (AFNCCF, 2019). Given adolescent’s tendency to seek help from peers first, these initiatives may be particularly valuable for this age group.

Finally, it is worth noting that one COVID-19 specific predictor, adherence to government guidance, was identified as a significant protective factor for both internalising difficulties and wellbeing. Whilst the reason for this is unclear, there is a potential that the families who adhered to the guidance were those who were more accepting and/or understanding of the COVID-19 pandemic, resulting in children feeling less anxiety or stress during this time. Interestingly, few other COVID-19 specific factors, such as having a parent who was shielding or lost their job, were significantly associated with children’s mental health outcomes. Thus, although largely null results were identified regarding these predictors, the absence of significant negative mental health outcomes for these groups of children could be considered a good sign, and still contributes to the evidence base regarding the COVID-19-related factors that are (or are not) a cause for concern.

### Limitations

There are several limitations of the present study that should be noted. In terms of the sample size, there were a relatively small number of participants (*n* = 290) limited to one regional area in England (the North West). Secondly, participants were also self-selecting, as the survey was sent home via their school, and they were free to choose whether to participate. Thus, there is potential that the findings were not representative of the experiences of this age group nationally. However, participant demographics were broadly reflective of national averages (Department for Education, 2021b) in terms of the proportion of young people eligible for FSM and those belonging to a BAME background. Third, the proportion of missing data was relatively high (12.8%). To account for this, an EM algorithm was used in order to reduce bias (Graham, 2012), and so this was not considered to be problematic. Fourth, it was not possible to include all possible candidate protective factors in the present study, and so some significant contributors to adolescent mental health outcomes may have been missed. Fifth, the internal consistency of the LOT-R measure of optimism was slightly lower than the widely agreed upon 0.7 cut-point, indicating that items may not have been reliable. Finally, the cross-sectional nature of this study limits the extent to which causation can be inferred. Thus, the direction of the relationships between the predictor and outcome variables cannot be confirmed.

It is also worth highlighting potential issues with the use of the term ‘protective factors’ in the present study. There is some contention in the resilience literature regarding the use of the terms protective and promotive factors; some suggest the use of the term ‘protective factors’ is only appropriate when examining the interaction term or moderating effect of factors on the relationship between risk factors and poorer mental health outcomes. Conversely, ‘promotive’ should be used for factors that are directly associated with positive outcomes, regardless of risk status (Luthar & Zelazo, 2003). As risk factors or interaction terms were not directly explored in the present study, there may be some question over whether protective factors were truly identified. However, Luthar et al. (2000) have suggested that the importance of interaction effects should not detract from the significance of main-effect associations, and the term ‘protective’ should be used in a broader sense, referring to all constructs linked with positive adaptation in at-risk groups. Arguably the COVID-19 pandemic has been a risk factor for all adolescents in terms of their mental health and wellbeing and, although they have not all had equal experiences, emerging evidence suggests that as a group they are at increased risk of developing mental health difficulties as a result of the pandemic (e.g., Loades et al., 2020). Nevertheless, in time, future research should seek to explore longitudinally the distinct promotive and protective factors that contributed to the onset of adolescent mental health difficulties for at-risk groups, as a direct consequence of the pandemic.

## Conclusion

The present study aimed to address a key gap in the present literature by examining protective factors across multiple domains for positive mental health outcomes amongst early adolescents, during the COVID-19 pandemic. Findings highlight the value of ensuring that young people have a positive experience during any future lockdowns, and the importance of identifying and intervening with any who have had negative experiences. Importantly, the role of optimism is highlighted as a protective factor in the face of adverse experiences that are beyond our control. Furthermore, results also emphasise the need to boost young people’s connections with others on the return to formal schooling, and during the easing of lockdown restrictions in the future. In summary, the ‘ordinary magic’ of supportive relationships and positive experiences appear to be some of the key factors needed to promote adolescents’ mental health and wellbeing and to help them overcome the difficulties posed by the COVID-19 pandemic. Thus, results mirror what Masten (2001, p. 235) first posited 20 years ago: “resilience does not come from rare or special qualities, but from the everyday magic of the ordinary, normative human resources in… children, in their families and relationships, and in their communities”.

## Footnotes

1 Schools that offer places based on children’s abilities, often requiring them to pass an entrance examination

2 It is notable that the bivariate correlation was negative (*r* = −0.10; Table 3), and the regression coefficient from Step 2 of the hierarchical regression (*β* = 0.02) was not statistically significant, indicating a likely case of statistical suppression. To identify the cause, variables were taken out of the Step 3 model one at a time. Fear about COVID-19 became a stronger, and statistically significant, positive predictor of wellbeing only when Optimism was included at Step 3. Accordingly we attach no interpretive significance to fear of COVID-19 as a predictor of wellbeing.

## Declarations

All procedures performed in studies involving human participants were in accordance with the ethical standards of the institutional and/or national research committee and with the 1964 Helsinki Declaration and its later amendments or comparable ethical standards. The study was approved by the Liverpool John Moores University Research Ethics Committee (20/NSP/037).
